# Supplementary material for: ARF1 prevents aberrant type I interferon induction by regulating STING activation and recycling
Source: Nat Commun. 2023 Nov 1;14:6770. doi: 10.1038/s41467-023-42150-4 (PMC10620153; doi:10.1038/s41467-023-42150-4)
Supplement: Supplementary file 1 — Supplementary Information [file 41467_2023_42150_MOESM1_ESM.pdf]

Supplementary Information

ARF1 prevents aberrant type I interferon induction by regulating STING activation and recycling

Maximilian Hirschenberger[1], Alice Lepelley[2], Ulrich Rupp[3], Susanne Klute[1], Victoria Hunszinger[1], Lennart Koepke[1], Veronika Merold[4], Blaise Didry-Barca[2], Fanny Wondany[6], Tim Bergner[3], Tatiana Moreau[2], Mathieu P Rodero[2], Reinhild Rösler[7], Sebastian Wiese[7], Stefano Volpi[8,9], Marco Gattorno[8], Riccardo Papa[8], Sally-Ann Lynch[10,11], Marte G. Haug[12], Gunnar Houge[13], Kristen M. Wigby[14,15], Jessica Sprague[16], Jerica Lenberg[15], Clarissa Read[3], Paul Walther[3], Jens Michaelis[6], Frank Kirchhoff[1], Carina C. de Oliveira Mann[4], Yanick J. Crow[2,5,*], Konstantin M.J. Sparrer[1,*]

[1]Institute of Molecular Virology, Ulm University Medical Center, 89081 Ulm, Germany
[2]Université Paris Cité, *Imagine* Institute, Laboratory of Neurogenetics and Neuroinflammation, INSERM UMR1163, F-75015, Paris, France
[3]Central Facility for Electron Microscopy, Ulm University, 89081 Ulm, Germany
[4]Institute of Virology, Technical University of Munich, 81675 Munich, Germany
[5]MRC Human Genetics Unit, Institute of Genetics and Cancer, University of Edinburgh, Edinburgh, United Kingdom
[6]Institute of Biophysics, Ulm University, 89081 Ulm, Germany
[7]Core Unit Mass Spectrometry and Proteomics, Ulm University Medical Center, 89081 Ulm, Germany
[8]Centro per le Malattie Autoinfiammatorie e Immunodeficienze, IRCCS Istituto Giannina Gaslini, Genoa, Italy
[9]Università degli Studi di Genova, Genoa, Italy
[10]Children's Health Ireland, Crumlin, Dublin, Eire
[11]University College Dublin, Dublin, Eire
[12]Department of Medical Genetics, St. Olav's Hospital, Trondheim, Norway
[13]Department of Medical Genetics, Haukeland University Hospital, 5021 Bergen, Norway
[14]Department of Pediatrics, Division of Genetics, University of California, San Diego, California, USA
[15]Rady Children's Institute for Genomic Medicine, San Diego, California, USA
[16]Division of Pediatric and Adolescent Dermatology, University of California, San Diego, California; Rady Children's Hospital, San Diego

*Address correspondence to: Yanick J. Crow: yanickcrow@mac.com; Konstantin M.J. Sparrer: Konstantin.Sparrer@uni-ulm.de.

Supplementary Figures 1 – 7

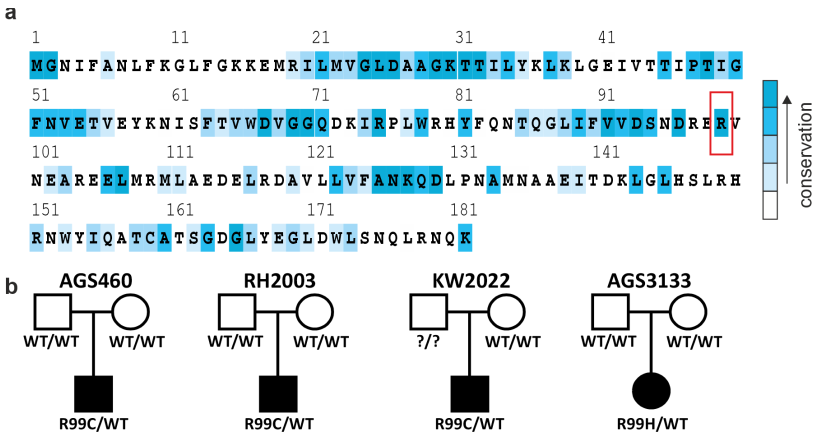

Supplementary Figure 1: Conservation of ARF1 and pedigree of patients. a, Conservation of the amino acids in ARF1 (blue, conserved; white, not conserved), with R99 highlighted in red as analysed by ConSurf. **b**, Pedigrees of the four patients ascertained with a substitution at R99 of ARF1.

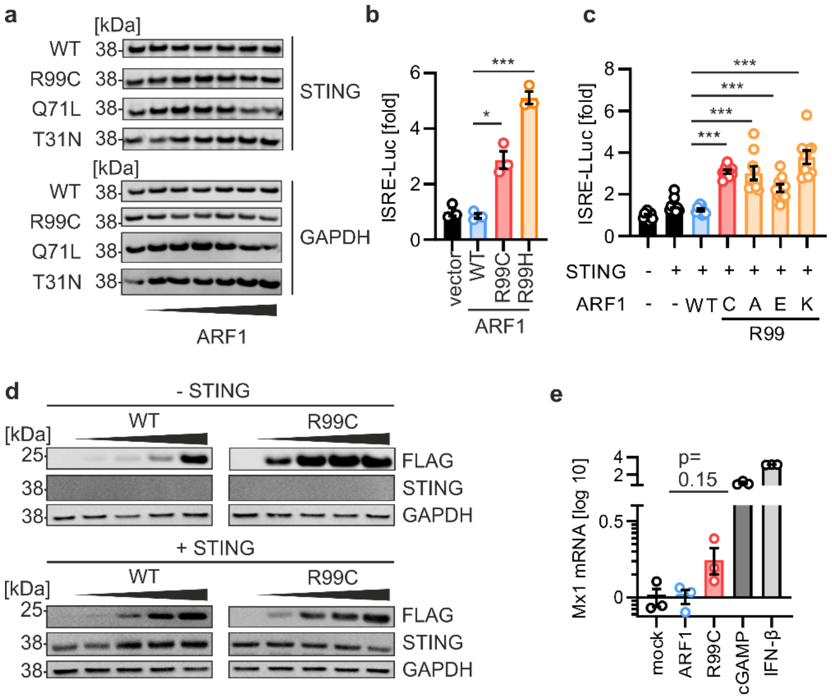

Supplementary Figure 2: Expression of ARF1 WT and ARF1 R99C. a, FLAG-tagged ARF1 WT, R99C, Q71L or T31N were transiently expressed in 293-Dual-hSTING-R232 reporter cells. Representative immunoblots of whole cell lysates (WCLs) showing the corresponding expression of STING and GAPDH, stained with anti-STING and anti-GAPDH. **b,** Impact of FLAG-tagged ARF1 WT, R99C and R99H expression by transient transfection on ISRE promoter activity in HEK293T cells co-expressing STING (+STING). ISRE-driven Firefly luciferase (Fluc) was quantified 32 h post transfection and normalised to GAPDH-promoter driven Renilla luciferase. Dots represent mean of n = 3 ± SEM (biological replicates). **c,** Impact of FLAG-tagged ARF1 WT, R99C, R99A, R99E and R99K expression by transient transfection on IFNB1 promoter activity in HEK293T cells co-expressing STING (+STING). IFNB1 promoter-driven firefly luciferase (Fluc) was quantified 24 h post transfection and normalised to HSV-thymidine kinase promoter-driven Renilla luciferase. Dots represent mean of n = 9 ± SEM (from 3 biological replicates). **d,** Exemplary immunoblot of WCLs of HEK293T cells transiently expressing FLAG-tagged ARF1 WT or R99C and co-expressing STING-FLAG (bottom) or empty vector (top). Blots were stained with anti-FLAG, anti-STING and anti-GAPDH. **e**, Impact of ARF1 WT or R99C expression on ISG induction in primary normal human lung fibroblasts (NHLF) as assessed by qPCR of Mx1 mRNA 72 h post transduction. IFN-β (1000 U/mL, 16 h) and cGAMP (10 µg/ml, 16 h) served as positive controls. Bars represent mean of n = 3 ± SEM (biological replicates). Statistical analysis was performed using a two-tailed Student's t test with Welch's correction (b, c, e). Significances are indicated as: *, $p < 0.05$; ***, $p < 0.001$. Exact P values and Source Data are provided as a Source Data file.

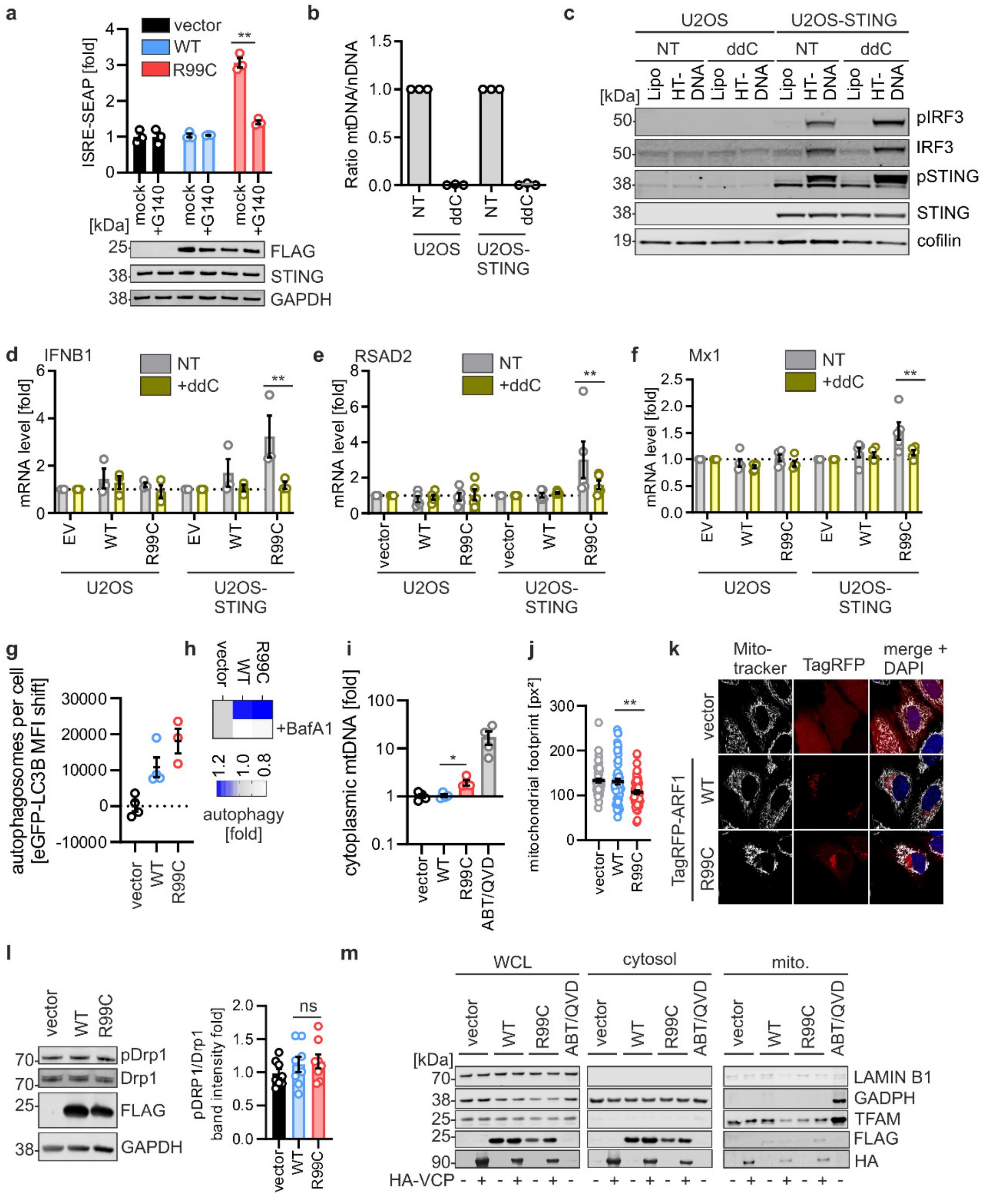

Supplementary Figure 3: Depletion of mtDNA reduces ISG induction in the presence of ARF1 R99C. a, Impact of transient transfection of FLAG-tagged ARF1 WT or R99C on ISRE promoter activity in 293-Dual-hSTING-R232 cells either mock treated or treated with G140 (2.5 µg/ml, 32 h). SEAP activity was quantified 32 h post transfection and normalised to cell viability. Bars represent mean of n = 3 ± SEM (biological replicates). Lower panel: Corresponding immunoblots of WCLs stained by anti-FLAG, anti-STING and anti-GAPDH. **b**, mtDNA depletion in U2OS and U2OS-STING cells assessed by qPCR for the mitochondrial gene MT-COXII and the nuclear gene GAPDH. mtDNA depletion was induced by treating the cells with 100 µM 2',3' dideoxycytidine (ddC) for seven to fourteen days. Bars represent mean of n = 3 ± SEM (biological replicates). **c**, Exemplary immunoblot of WCLs of U2OS and U2OS-STING cells left untreated (NT) or treated with 100 µM ddC for seven to fourteen days

and subsequently stimulated with HT-DNA (2 µg/mL, 4 h) or treated with lipofectamine (Lipo) only. Blots were stained with anti-pIRF3, anti-IRF3, anti-pSTING, anti-STING and anti-cofilin. **d-f**, Impact of ARF1 WT, R99C or empty vector (EV) expression on ISG induction in U2OS and U2OS-STING cells treated with 100 µM ddC for seven to fourteen days or left untreated (NT). mRNA levels of Mx1, IFNB1 and RSAD2 were assessed by qPCR 24 h post transfection. Bars represent mean of n = 3 (d), n = 5 (e, f) ± SEM (biological replicates). **g**, Impact of ARF1 WT and ARF1 R99C on autophagosome levels. HEK293T cells stably expressing eGFP-LC3B were transfected with TagRFP-ARF1 WT, TagRFP-ARF1 R99C or empty vector control. Shifts in autophagosome levels were assessed using flow cytometry. n = 4 (vector, WT) , n = 3 (R99C) ± SEM (biological replicates). **h**, Impact of ARF1 WT and ARF1 R99C on autophagosome levels in the presence and absence of Bafilomycin A1. HEK293T cells stably expressing eGFP-LC3b were transfected with TagRFP-ARF1 WT, TagRFP-ARF1 R99C or empty vector control. Shifts in autophagosome levels were assessed using flow cytometry. n = 4 ± SEM (biological replicates **i**, qPCR of mtDNA (MT-D-Loop) in the cytosolic fraction of ARF1 WT, R99C, Q71L and vector transfected HEK293T ATG5 KO relative to total normalized cellular mtDNA (mtDNA/nuclear DNA) using the ΔΔCT method. n = 4 ± SEM. **j**, Analysis of the mitochondrial footprint from the images shown in (**k**) using the MiNA plugin (StuartLab) for ImageJ (Fiji). Lines represent mean of n = 62 (vector), n = 53 (WT), n = 51 (R99C) ± SEM (individual cells). **k**, Exemplary live cell confocal laser scanning microscopy images of HeLa cells expressing TagRFP-tagged ARF1 WT, R99C or vector control. Cells were treated with Mitotracker (1 µM) for 30 min at 37°C. Nuclei, Hoechst 33342 (blue). Scale bar, 10 µm, **l**, Exemplary immunoblot of WCLs of HEK293T cells transiently expressing ARF1 WT, R99C or vector control. Blots were stained with anti-pDRP1, anti-DRP1, anti-FLAG and anti-GAPDH. Quantification of the band intensities for pDRP1 normalized to the band intensities of DRP1. Bars represent mean of n = 8 ± SEM (biological replicates). **m,** Exemplary immunoblots showing fractionation of HEK293T cells expressing ARF1 WT, R99C or vector control as well as VCP. WCLs and fraction blots stained by anti-FLAG, anti-HA, anti-TFAM (mitochondria), anti-LAMIN B1 (nucleus) and anti-GAPDH (cytosol). Statistical analysis was performed using a two-tailed Student's t test with Welch's correction (a, i, j, m) or two-way ANOVA (d, e, f). Significances are indicated as: *, $p < 0.05$; **, $p < 0.01$; ***, $p < 0.001$. Exact P values and Source Data are provided as a Source Data file.

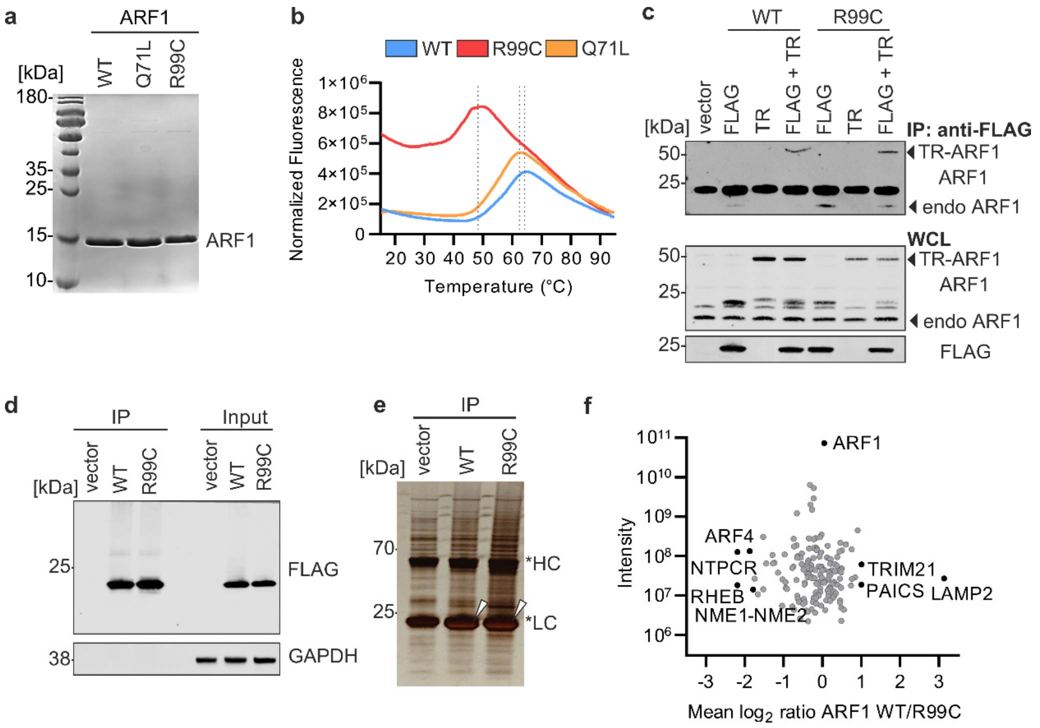

Supplementary Figure 4: Characterisation of stability and dimerization of ARF1 WT and R99C. a, Coomassie stained SDS-PAGE gel of purified recombinant human ARF1 WT, Q71L and R99C proteins lacking its N-terminal 17 amino acids. **b,** Thermal shift assay of ARF1 WT, Q71L and R99C. Respective inflection temperatures are: ARF1 WT 58.3 °C; ARF1 R99C 44.4 °C; ARF1 Q71L 56.8 °C. Data are representative of two biological replicates. **c,** Immunoprecipitation (IP) of FLAG-tagged ARF1 WT and R99C by anti-FLAG-beads. HEK293T cells were transfected with empty vector, FLAG-tagged ARF1 WT or R99C (FLAG), or TagRFP(TR)-tagged ARF1 WT or R99C, or with FLAG-tagged ARF1 WT and TagRFP-tagged ARF1 WT or FLAG-tagged ARF1 R99C and TagRFP-tagged ARF1 R99C (FLAG+TR). 24 h post transfection, cells were harvested and anti-FLAG IP was performed. Blots were stained with anti-ARF1 and anti-FLAG. **d-e,** IP of FLAG-tagged ARF1 WT and R99C by anti-FLAG-beads, from cell lysates of HEK293T cultivated in SILAC light medium or heavy medium for five passages, then transfected with empty vector, FLAG-tagged ARF1 WT or ARF1 R99C, harvested and lysed 24 h post transfection. After electrophoresis, in (**d**) western blot was performed and stained with anti-FLAG and anti-GAPDH, in (**e**) gel was stained with SDS-Gels silver staining. HC: heavy chain, LC: light chain. **f,** Scatter plot showing Intensity versus mean log2 ratio between heavy and light chain (ARF1 WT versus R99C sample). Highly regulated genes and ARF1 are labelled. Data from Supplementary Data 2.

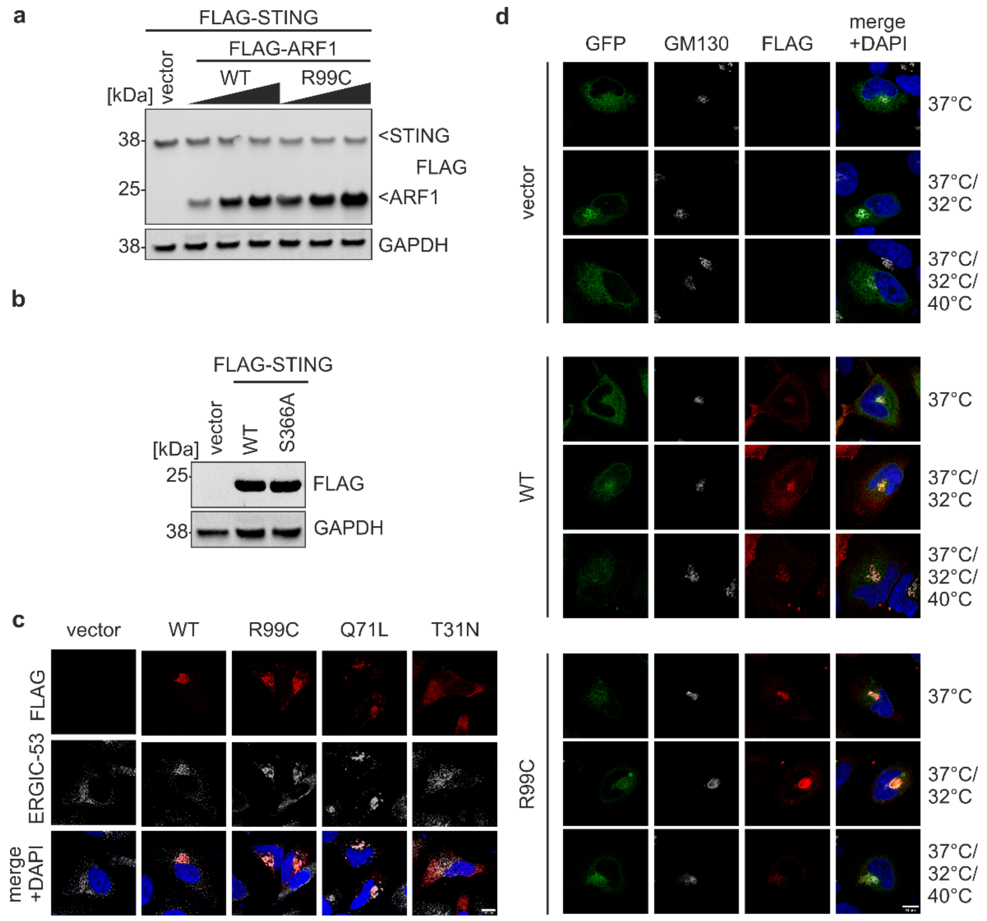

Supplementary Figure 5. ARF1 mediated ERGIC morphology and retrograde transport from the ERGIC/Golgi to the ER. a, FLAG-tagged ARF1 WT and R99C were transiently expressed in HEK293T cells co-expressing STING-FLAG, ISRE-promoter controlled Firefly luciferase and GAPDH-promoter controlled Renilla luciferase. Representative immunoblots of WCLs showing the corresponding expression of ARF1 WT, R99C, STING and GAPDH, stained with anti-FLAG and anti-GAPDH. **b,** Representative immunoblot of WCLs for Fig. 5c. Blots were stained with anti-FLAG and anti-GAPDH. **c,** Exemplary confocal laser scanning microscopy images of Hela cells expressing FLAG-tagged ARF1 WT, R99C, Q71L or T31N, corresponding to Fig. 5g. Cells were stained 24 h post transfection with anti-FLAG (red) and anti-ERGIC-53 (grey). Nuclei, DAPI (blue). Scale bar, 10 µm. **d,** Exemplary confocal laser scanning microscopy images of HeLa cells expressing VSVG-ts045-KDELR-eGFP (green) and indicated FLAG-tagged ARF1 mutants, corresponding to Fig. 5h. Cells were incubated at 37°C for 24 h and the different temperature shifts (37°C/32°C/40°C) as indicated. Staining with anti-FLAG (red) and anti-GM130 (grey). Nuclei, DAPI (blue). Scale bar, 10 µm.

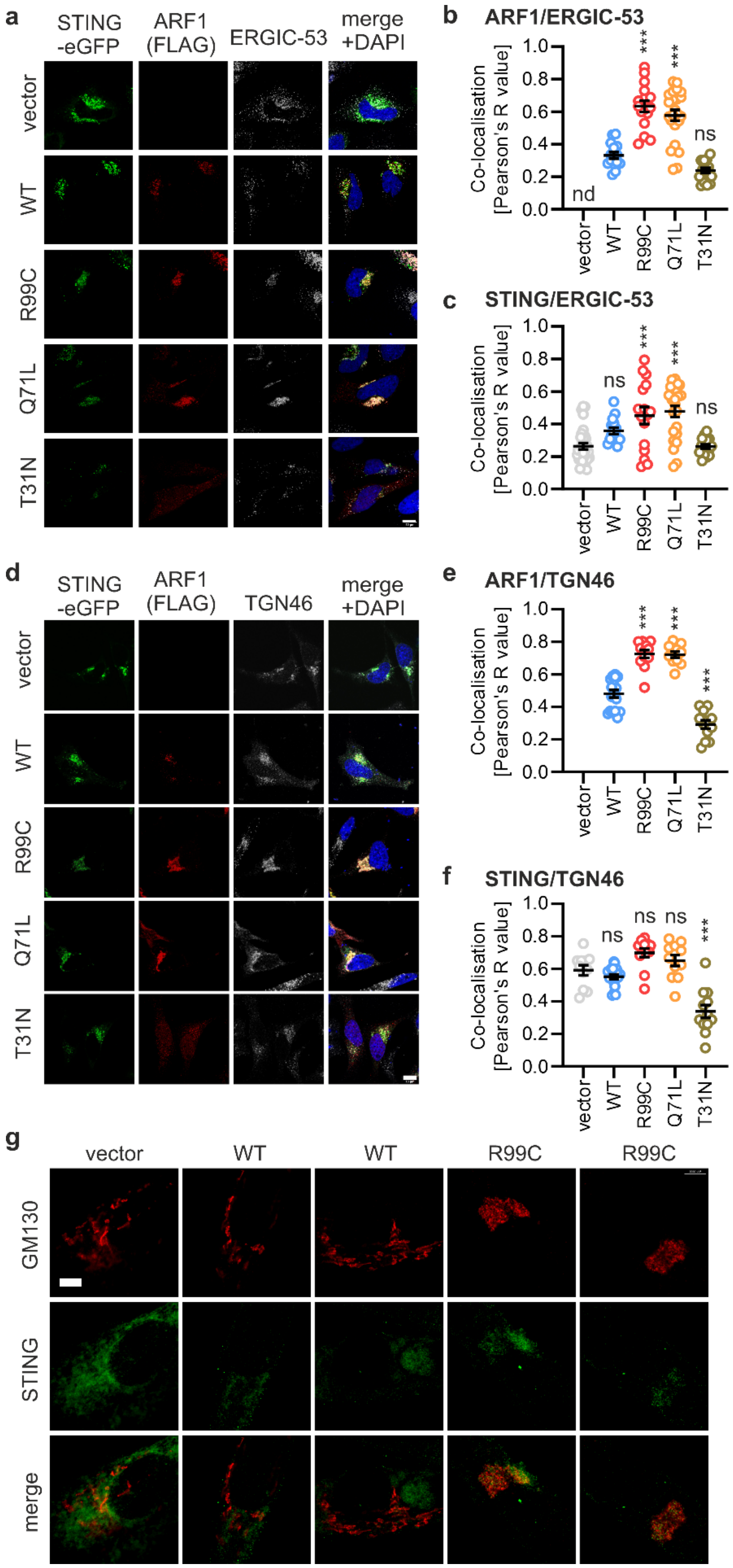

Supplementary Figure 6: ARF1 R99C has increased localisation at the cis- and trans-Golgi. a, Exemplary confocal laser scanning microscopy images of STING-eGFP (green) and indicated FLAG-tagged ARF1 mutants in HeLa cells. Cells were stained 24 h post transfection with anti-FLAG (red) and anti-ERGIC-53 (grey). Nuclei, DAPI (blue). Scale bar, 10 µm, **b,**

Quantification of the co-localisation of ARF1 and ERGIC-53 and **c**, STING and ERGIC-53 from the images shown in (**a**) using Pearson's correlation coefficient. Lines represent mean of n = 31 (vector), n = 15 (WT), n = 16 (R99C), n = 23 (Q71L), n = 16 (T31N) ± SEM (individual cells). **d,** Exemplary confocal laser scanning microscopy images of STING-eGFP (green) and indicated FLAG-tagged ARF1 mutants in HeLa cells. Cells were stained 24 h post transfection with anti-FLAG (red) and anti-TGN46 (grey). Nuclei, DAPI (blue). Scale bar, 10 µm. **e**, Quantification of the co-localisation of ARF1 and TGN46 and **f**, STING and TGN46 from the images shown in (**d**) using Pearson's correlation coefficient. Lines represent mean of n = 11 (vector), n = 18 (WT), n = 12 (R99C), n = 11 (Q71L), n = 12 (T31N) ± SEM (individual cells). **g,** Exemplary STED super-resolution microscopy images of NHLF cells transduced with lentiviruses expressing indicated ARF1 constructs or empty vector. 48 h post transduction the cells were stained with anti-STING (green), anti-FLAG (not shown) and anti-GM130 (red). Only FLAG-staining positive cells are displayed. Scale bar, 5 µm. Statistical analysis was performed using a two-tailed Student's t test with Welch's correction (b, c, e, f). Significances are indicated as: ***, $p < 0.001$. Exact P values and Source Data are provided as a Source Data file.

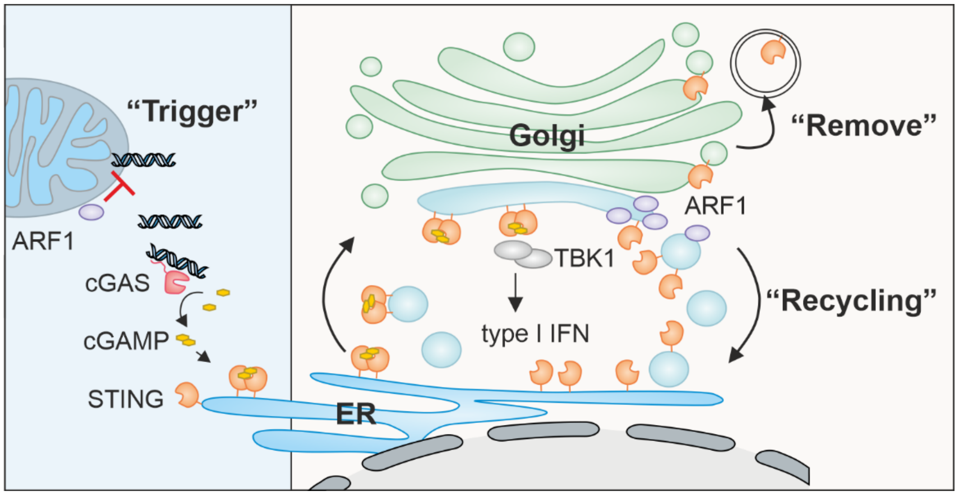

Supplementary Figure 7: Model summarizing the involvement of ARF1 in cGAS-STING signal triggering and termination.

Supplementary patient information

AGS460. This male was born to unrelated parents with no family history of note. He was delivered at 40 weeks gestation after a normal pregnancy, with a birth weight of 3.05kg and head circumference of 35cm. He was noted to have a cleft palate and cryptorchidism, both requiring surgical correction. A karyotype was normal. Skin disease was obvious from the first month of life, particularly involving the hands and feet, but also the ears, cheeks, nose, elbows, knees and heels. These lesions were painful and itchy, and exacerbated by cold with definite improvement in the summer months, leading to a diagnosis of recurrent chilblains. Initially at least, exacerbation of these lesions was accompanied by fever. There has been a marked ulcerative aspect to his skin disease, with frank tissue loss, resulting in major difficulties with walking and hand use due to pain. Significant psychomotor delay was obvious from age 14 months, with sitting at 16 months of age, walking independently at age 22 months, and an absence of the development of any useful speech. There are no focal neurological signs, no pyramidal or extrapyramidal features and he has never experienced seizures. Brain MRI at age 9 years showed a thin corpus callosum and mild reduction of posterior white matter, with no evidence of periventricular nodular heterotopia. No calcification was noted on cerebral CT scan, and interferon alpha activity in the cerebrospinal fluid was normal (both assessed at 9 years of age). Autoantibodies (anti-dsDNA, ENA, ANCA, ANA) were negative. His growth has been poor (height 146cm, weight 33kg, both < 0.4th centile at age 15 years). He is now aged 20 years. His neurological condition remains stable with no signs of regression. There has been an improvement in his skin disease since puberty. A 6 interferon stimulated gene (ISG) interferon score at age 14 years was markedly elevated at 87 (normal < 2.46), and again elevated (4.22, normal < 2.7) aged 17 years. He was shown to harbour a *de novo* p.(Arg99Cys) mutation in ARF1.

RH2003. This male was born to unrelated parents with no family history of note. He was delivered at 40 weeks gestation after a normal pregnancy, with a birth weight of 3.3kg and head circumference of 37.5cm (-1.96 SD) at age 2 months. He demonstrated a delay in the acquisition of skills, walking at age 2 years 9 months. His gait has remained somewhat unsteady and there is a tendency to toe walk. He was subsequently diagnosed with moderate to severe intellectual disability with autistic features. He has no words but can communicate using assistive technology. He experienced febrile seizures as a child, but has not demonstrated any epilepsy. Cerebral MRI at age 13 years was normal, with no periventricular nodular heterotopia. Vision and hearing are unremarkable. Now aged 18 years he lives in a care home with full-time supervision. Skin involvement was present from early childhood. He was seen by a dermatologist at age 15 years due to marked exacerbation of his skin disease over a period of a few months. He had increasing difficulty using his hands and feet properly due to the severity and pain of his skin disease, which was particularly marked over the dorsum of the hands and feet but also involved his ears. On examination there was a confluent maculo-papular rash with red-blue discolouration, and a central crust with some evidence of ulceration. Blistering has been observed on occasion, and the rash is notably worse in cold weather. A skin biopsy was reported to be consistent with chilblain lupus erythematosus. Local steroid treatment and heated gloves led to some clinical improvement. Blood count, inflammatory markers and autoantibody screen were normal, as was screening for porphyria. Interferon signalling status has not been assessed. He was shown to harbour a *de novo* p.(Arg99Cys) mutation in ARF1.

KW2022. This male was born to unrelated parents. His mother had a history of a language-based learning disability and febrile seizures as a child. He was delivered at 34 weeks gestation, following a pregnancy complicated by late prenatal care, exposure to carbegoline (due to a maternal history of hyperprolactinemia), and premature rupture of membranes. He was delivered vaginally and was treated empirically for sepsis and feeding difficulties in hospital for two weeks. Birthweight was 2.27 kg, length 48 cm and head circumference 31 cm. He failed a new-born hearing screen, and subsequent testing revealed a unilateral sensorineural hearing loss requiring a hearing aid. He has demonstrated non-progressive development delay, acquiring his first words aged 2 years and walking at 27 months of age. He has an expressive language delay and uses a communication device. MRI brain and auditory nerves were normal, and a sleep deprived EEG revealed only rare epileptiform discharges and no clinical seizures. His head circumference at age 3 years was 47.6cm. At approximately 1 year of age he developed an erythematous macular papular rash involving the hands, feet, lower legs and ears, which was considered as pernio-like following a dermatology assessment. Biopsy of a fresh lesion revealed an interface dermatitis with a histiocytic/neutrophilic infiltrate. There was no improvement following a trial of topical steroids. The lesions continue to flare intermittently, and are notably exacerbated by cold weather and intercurrent illness. Screening laboratory studies obtained as part of the evaluation for his rash revealed elevated transaminases in the absence of anomalies on liver imaging and in the face of a normal liver biopsy. Interferon signalling status has not been assessed. He was shown to harbour a p.(Arg99Cys) mutation in ARF1. His mother was wild-type at this amino acid, while paternal DNA was not available.

AGS3133. This female was born to unrelated parents with no family history of note. She was delivered at 40 weeks gestation after a normal pregnancy, with a birth weight of 3.46kg. She was identified to have a ventricular septal defect, subsequently requiring surgical correction at age 11 years. She exhibited severe developmental delay from an early age, with no evidence of regression. Now aged 16 years she can stand with support, but cannot walk independently and uses a wheelchair. There is no spasticity and there are no extrapyramidal movements. She has stereotypies including hand wringing and rocking and has episodes of hyperventilation. She has never experienced any seizures. She has no speech, babbles and is doubly incontinent. Her head circumference was 52cm at age 15 years (0.4th - 2nd centile), and weight 44kg (2nd centile). She demonstrates poor muscle bulk, hypotonia and rocker-bottom feet. She has a narrow palate with a double row of teeth in the lower jaw and prominent teeth in the upper jaw. Her hands and feet are petite, and her feet are cold but with no history of chilblains. There is a haemangioma on her back, and two largish café au lait patches. She entered puberty as normal, her general health is good and she is on no medications. Cranial MRI aged 18 months was normal except for delayed myelination, with no evidence of periventricular nodular heterotopia. Further scanning aged 12 years revealed normal myelination. A 24 ISG interferon score was elevated (3.685; normal < 2.74) at age 15 years. She was shown to harbour a *de novo* p.(Arg99His) mutation in ARF1.
