# Supplementary material for: ARF1 prevents aberrant type I interferon induction by regulating STING activation and recycling
Source: Nat Commun. 2023 Nov 1;14:6770. doi: 10.1038/s41467-023-42150-4 (PMC10620153; doi:10.1038/s41467-023-42150-4)
Supplement: Supplementary file 2 — Description of Additional Supplementary files [file 41467_2023_42150_MOESM2_ESM.pdf]

Description of Additional Supplementary files

File name: Supplementary Data 1
Description: Molecular Data relating to ARF1

File name: Supplementary Data 2
Description: Raw data of the SILAC mass spectrometry

File name: Supplementary Data 3
Description; PantherDB Analysis
